# Ruptured Large Gastrointestinal Stromal Tumor: A Case Report and Review of Literature

**DOI:** 10.1155/2023/2733295

**Published:** 2023-08-16

**Authors:** Javereeya Abdul Jabbar, Amr Elmekresh, Yousif Eltayeb

**Affiliations:** ^1^Dubai Health Authority, Dubai, UAE; ^2^Dubai Medical College for Girls, Dubai, UAE

## Abstract

Gastrointestinal stromal tumors (GIST) account for the majority of non-epithelial, mesenchymal tumors occurring in the gastrointestinal tract. Usually, the tumor measures a few centimeters, and cases larger than 15 cm are rare. Here, we report a rare case of a previously healthy 50-year-old woman, with generalized abdominal pain and increased abdominal girth for over nine months. Imaging showed a very large cystic lesion (21 cm × 15 cm × 24 cm) arising from the greater curvature of the stomach with rupture of the lesion into the intraperitoneal space. The patient was taken for exploratory laparotomy, which revealed a ruptured large cystic mass (21 cm × 15 cm × 24 cm) occupying the upper abdomen and encompassing the greater curvature of the stomach, body, and tail of the pancreas, as well as part of the spleen.

## 1. Introduction

A gastrointestinal stromal tumor (GIST) is known to be the most common mesenchymal tumor that tends to arise anyplace in the alimentary canal, accounting for almost 60% of all gastric stromal tumors [1, 2]. According to the National Institutes of Health guidelines, GISTs are classified per their size, location, and degree of mitoses [3].

A review of published literature reported tumor sizes ranging from 1.0 to 10 cm, with a few cases of large masses measuring up to 15.5 cm [3, 4]. Here, we report a rare case of a very large ruptured GIST measuring 21 cm × 15 cm × 24 cm in a patient aged 50 years.

## 2. Case Report

A previously healthy 50-year-old lady presented to the Emergency Department, Rashid Hospital, Dubai, ten minutes after a fall sustaining trauma to her abdomen. The patient had noticed gradual progressive abdominal distention over the preceding nine months. It was associated with abdominal pain and discomfort initially on the left side followed by generalized abdominal pain. Furthermore, she experienced fresh bright bleeding per rectum that lasted a month. She noted an unintentional mild loss of weight over the same period. According to her personal history, she quit smoking ten years before and is a social drinker. The family history was positive for colon cancer in her mother.

On physical examination, the abdomen was grossly distended and tense mainly in the central abdomen, without any significant guarding or rigidity. Her initial laboratory tests on presentation to the emergency department were as follows: hemoglobin 10.4 g/L, C-reactive protein 5.3 mg/L, lactic acid 3.1 mmol/L down-trending to 1.6 mmol/L after hydration, procalcitonin 0.07 ng/mL, urea 48 mg/dL, amylase 109 U/L and lipase 80 U/L. Tumor markers were within normal limits; cancer antigen 125 8.8 U/mL and Ca 19–9 8.8 U/mL.

**Figure 1:**
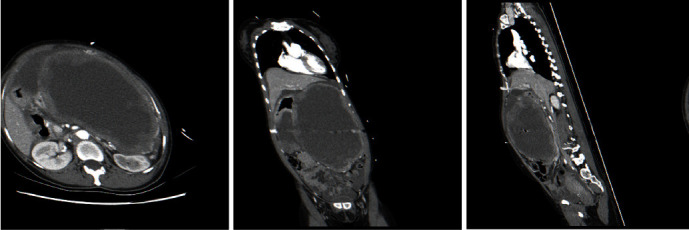
CT of the chest/abdomen/pelvis with contrast showed a large predominantly cystic lesion with its epicenter in the peritoneal cavity, its wall has nodular thickening and heterogenous peripheral enhancement with central cystic/necrotic area. Free fluid (25HU) was also appreciated in the abdomen and pelvis. A significant mass effect was seen on the retroperitoneal structures as well as the stomach and bowel loops. The mass measures 21 cm × 15 cm × 24 cm and is in direct contact with the greater curvature of the stomach.

Computed tomography (CT) of the chest/abdomen/pelvis with contrast showed a large predominantly cystic lesion with its epicenter in the peritoneal cavity. Its wall has nodular thickening and heterogenous peripheral enhancement with a central cystic/necrotic area. Free fluid (25HU) was also appreciated in the abdomen and pelvis. A significant mass effect was seen on the retroperitoneal structures as well as the stomach and bowel loops. CT findings were consistent with the rupture of the mass with the spillage of its contents into the intraperitoneal space. The mass measures 21 cm × 15 cm × 24 cm (transverse × AP × CC) and was in direct contact with the greater curvature of the stomach. The pancreas was significantly pushed post-inferiorly and to the right but with no definite invasion of the mass to the pancreatic parenchyma. Furthermore, two other masses were in the pelvis on either side representing uterine fibroids. The radiology report gave a diagnosis of GIST arising from the greater curvature of the stomach with the rupture to the intraperitoneal space. The two lesions in the pelvis likely represent uterine fibroids (Figure 1).

Exploratory laparotomy was performed, which showed a ruptured large cystic mass occupying the upper abdomen encompassing the greater curvature of the stomach, body, and tail of the pancreas, as well as part of the spleen. The site of perforation of the mass was at the inferior pole with spillage of necrotic debris mixed with blood-tinged fluid. Resection of the mass was performed along with partial resection of the stomach (sleeve), distal pancreatectomy, and splenectomy along with part of the transverse colon. The colonic anastomosis was delayed for a relook. Peritoneal fluid, resected cyst, and the part of the transverse colon were sent for histopathology.

Two days following the initial procedure, the patient underwent a relook laparotomy, and the colon was anastomosed. The peritoneal fluid result was reported as hemorrhagic and negative for malignant cells. The intra-abdominal cyst was identified as gastric GIST with high-risk spindle cells, pT4N0M0, arising from the stomach and infiltrating massively gastric wall and pancreas, with massive necrosis (in around 60% of tumor mass) and extensive hemorrhage. The gastric margin was free of tumors. Scanty viable pancreatic tissue was identified at the edge of the tumor mass.

**Figure 2:**
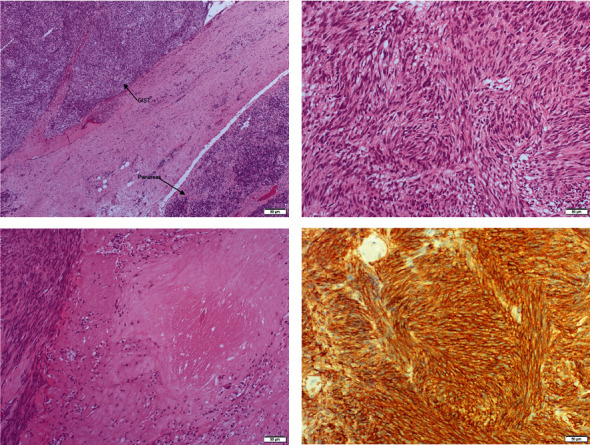
Immunohistochemistry study revealed tumor cells that were positive for DOG1, CD117, and CD34 and negative for S-100, SMA, and Desmin. Ki 67 was positive in 14% of the tumor cells—images and reports obtained from Dubai Health Authority Pathology Lab.

An immunohistochemistry study revealed tumor cells that were positive for DOG1, CD117, and CD34 and negative for S-100, SMA, and Desmin. Ki 67 was positive in 14% of the tumor cells (Figure 2). Additional resected specimens, such as the spleen and part of the transverse colon showed marked ischemic changes with severe necrosis, but no tumor infiltration was identified. The case was discussed in a multi-disciplinary team meeting and decided to be followed up in the oncology department to start chemotherapy. She was further planned for adjuvant Imatinib for three years.

Following discharge, the patient was seen in the surgical outpatient clinic a month later for a follow-up. She was feeling well and able to tolerate small amounts of oral diet and on subsequent visits, there was much improvement. She received her post-splenectomy vaccines and was referred to the gynecology department given her uterine fibroids and is currently doing well.

## 3. Discussion

GISTs are relatively rare solid tumors, accounting for less than 5% of all alimentary tract tumors [1]. Recent advances and improvements in microscopic imaging and immunohistochemistry have individualized GIST as a separate tumor [4]. Such developments in scientific technology have led to the understanding of these tumors originating from the interstitial cells of Cajal, which physiologically express CD117 that further encodes a receptor of tyrosine kinase. However, CD117 is also expressed by multiple other tumors. Another immunohistochemical marker highly expressed is CD34, especially in esophageal and colorectal tumors.

GIST tumors more than often, are asymptomatic, incidentally diagnosed during imaging, or evaluation, and are usually less than 2 cm. However, the patient presented in this case noticed abdominal distension for nine months occupying the greater curvature of the stomach, body, and tail of the pancreas and part of the spleen. North England review of cancer patients, identified a total of 42 tumors with a median age of being of 68 years. The majority of these tumors originated in the stomach (85.7%), with a mean tumor size of 5.46 cm [4].

Tumors larger than 15 cm are rare and only a few cases are reported mainly in older people. Wang et al. reported the case of a 74-year-old woman who had continuous, worsening abdominal pain for a few days. A CT abdomen demonstrated hypoechoic intraperitoneal mass measuring 15.6 cm × 14.7 cm × 13.6 cm. Exploratory laparotomy was performed, and the tumor was found to have necrosis and cystic degeneration without splenic or nodal invasion [1]. Similarly, Qian et al. reported the case of a 67-year-old male with a history of benign prostatic hyperplasia who was incidentally diagnosed with GIST at the right iliac fossa measuring 11.3 cm × 12.7 cm × 14 cm. The patient was asymptomatic but had a significant history of chronic metabolic diseases including diabetes, hypertension, and chronic kidney disease [3].

Histopathological examination of the cases included in the literature showed morphologically identified spindle-shaped cells. Immunohistochemistry demonstrated (+) CD117. However, Wang's case also showed (+) H-Caldesmon and Ki67 in 5% of tumor cells [4, 5]. Similarly, our case was also positive for CD117, CD34, DOG1, and Ki67 being positive in 14% of the tumor cells.

Large tumors can cause mass effects and give symptoms like obstruction or compression of the gastrointestinal tract leading to early satiety, change in bowel habits, dysphagia, or jaundice. Rupture of the tumor can lead to internal bleeding and peritonitis [6]. This is highlighted in a case of a 71-year-old male who presents with complaints of diffuse abdominal pain associated with periodic hematochezia for more than 24 hours. Physical examination revealed a tender abdomen. The ruptured mass was seen on CT measuring 5.2 cm × 4.1 cm × 3.0 cm. Moreover, in this report, the patient also had a metastatic groin mass with histopathology and immunohistochemistry reports characteristic of GIST findings [2].

Furthermore, these tumors do tend to invade and metastasize, most commonly in the abdominal cavity and liver [1]. Although the tumor size in our patient was extensive, we did not observe an invasion of remote organs or lymph nodes.

## 4. Conclusion

GISTs are relatively rare solid tumors, accounting for less than 5% of all alimentary tract tumors. Diagnosis is usually made incidentally during imaging for evaluation of other conditions. The tumor usually measures a few centimeters and cases larger than 15 cm are rare. Large tumors can cause mass effects leading to compression symptoms presenting as early satiety, change in bowel habits, dysphagia, jaundice, or bowel obstruction. Management of these tumors should always be discussed to identify cases with malignant potential for chemotherapy and adjuvant Imatinib.

## Data Availability

All data regarding this case report has been reported in the manuscript. Data supporting this research article are available from the corresponding author or first author upon reasonable request.

## References

[1] Wang L., Liu L., Liu Z., Tian Y., Lin Z. (2017). Giant gastrointestinal stromal tumor with predominantly cystic changes: a case report and literature review. *World Journal of Surgical Oncology*.

[2] Yuan Y., Ding L., Tan M., Han A. J., Zhang X. (2021). A concealed inguinal presentation of a gastrointestinal stromal tumor (GIST): a case report and literature review. *BMC Surgery*.

[3] Qian X. H., Yan Y. C., Gao B. Q., Wang W. L. (2020). Prevalence, diagnosis, and treatment of primary hepatic gastrointestinal stromal tumors. *World Journal of Gastroenterology*.

[4] McDonnell M. J., Punnoose S., Viswanath Y. K. S., Wadd N. J., Dhar A. (2017). Gastrointestinal stromal tumours (GISTs): an insight into clinical practice with review of literature. *Frontline Gastroenterology*.

[5] Ashoor A. A., Barefah G. (2020). Unusual presentation of a large GIST in an extraintestinal site: a challenging diagnosis dilemma. *BML Case Reports*.

[6] Parab T. M., DeRogatis M. J., Boaz AM A. M., Grasso S. A., Issack P. S., Duarte D. A. (2018). Gastrointestinal stromal tumors: a comprehensive review. *Journal of Gastrointestinal Oncology*.

